# Viral Membrane Channels: Role and Function in the Virus Life Cycle

**DOI:** 10.3390/v7062771

**Published:** 2015-06-23

**Authors:** Ching Wooen Sze, Yee-Joo Tan

**Affiliations:** 1 Department of Microbiology, Yong Loo Lin School of Medicine, National University Health System (NUHS), National University of Singapore, MD4, Level 3, 5 Science Drive 2, Singapore 117597, Singapore; E-Mail: micscw@nus.edu.sg; 2 Institute of Molecular and Cell Biology, Agency for Science, Technology and Research (A * STAR), Singapore 138673, Singapore

**Keywords:** viroporin, cytopathic effect, viral channel

## Abstract

Viroporins are small, hydrophobic trans-membrane viral proteins that oligomerize to form hydrophilic pores in the host cell membranes. These proteins are crucial for the pathogenicity and replication of viruses as they aid in various stages of the viral life cycle, from genome uncoating to viral release. In addition, the ion channel activity of viroporin causes disruption in the cellular ion homeostasis, in particular the calcium ion. Fluctuation in the calcium level triggers the activation of the host defensive programmed cell death pathways as well as the inflammasome, which in turn are being subverted for the viruses’ replication benefits. This review article summarizes recent developments in the functional investigation of viroporins from various viruses and their contributions to viral replication and virulence.

## 1. Introduction

From a biological standpoint, viruses are infectious genetic entities that can only replicate inside a living organism. Due to their relatively small genome sizes, viruses hijack and reprogram the host cellular pathways to facilitate their propagation. Viruses are classified into two general categories based on the type of genetic materials they carried, either RNA or DNA viruses. These viral genomes encode structural proteins for virion formation as well as enzymatic and accessory proteins to aid in infection and replication. One common type of accessory protein encoded by most viruses is the viral ion channel protein, viroporin. Viroporins are a class of small pore-forming proteins that have been shown to aid in multiple stages of the viral life cycle, from the initial genome replication to the final viral release stage (for a more recent review on viroporins, see [1,2,3,4,5,6]). Viroporins have at least one trans-membrane domain (TMD) and sometimes an extracellular membrane region that interacts with viral or host proteins. The hydrophobic regions of the proteins are capable of forming aqueous pores in the host lipid bilayer upon oligomerization. These pores could be ion-selective with a controlled gating mechanism, or non-selective ones that permeabilize the membrane. A cluster of basic residues within the viroporin aids in membrane insertion by interacting with the negatively charged phospholipids. The first and most extensively studied viroporin, M2 of influenza A virus (IAV), was identified in 1992 [7]; since then, several viral ion channel proteins have been discovered in other pathogenic animal viruses, including Hepatitis C virus (HCV), Human immunodeficiency virus (HIV)-1, and Coronaviruses (CoV) (Table 1).

**Table 1:** List of known viroporins and their known function in viral life cycle.

Virus	Viroporin	Amino Acid	Function in Viral Life Cycle	References
IAV	M2	97	Genome uncoating Glycoprotein processing/trafficking Delay protein trafficking through TGN Viral release	[8,9,10,11,12,13,14,15,16]
HIV-1	Vpu	77–86	Degradation of CD4 and trafficking of Env proteins Viral release	[17,18,19,20,21,22,23]
HCV	P7	63	Viral morphogenesis Viral polyprotein processing	[24,25,26,27]
CoV	E 3A	76 274	Viral morphogenesis and assembly Viral release	[28,29,30,31,32]
Poliovirus	2B 3A	97 87	Blocks ER-Golgi traffic/host protein secretion	[33,34]
Alphavirus/Sindbis virus	6K	60	Viral release	[35,36,37]
Coxsakievirus	2B	99	Inhibit protein trafficking through Golgi Induce apoptosis for viral release	[38,39,40,41]
Rotavirus	NSP4	175	Induce autophagy for viral protein transport	[42,43]
SV40	VP2 VP3 VP4	352 234 125	Translocation of DNA genome from ER to cytosol Viral release	[44,45,46,47,48]

**Figure 1:**
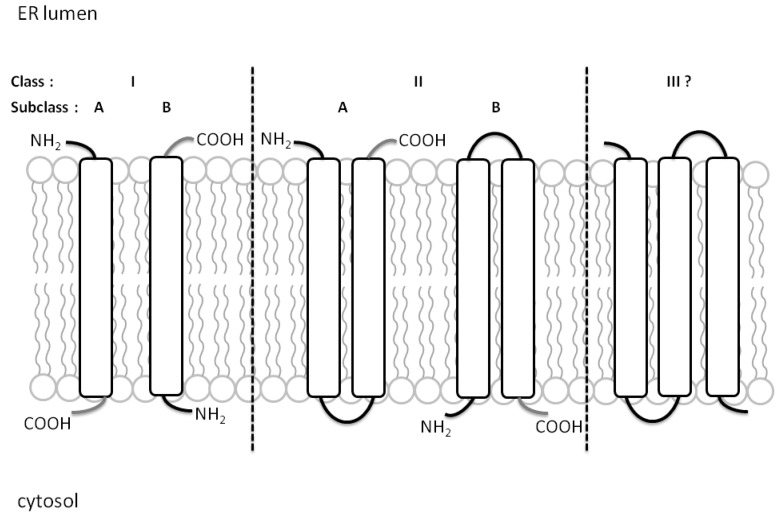
Classification of viroporins based on their membrane topology. Class I and Class II viroporins have one and two TMD, respectively. Class IA viroporins have their N-termini facing the ER lumen while Class IB have their C-termini in the cytosolic side. Class IIA viroporins have both the N- and C-termini in the lumenal side while Class IIB have them facing the cytosol. A putative Class III viroporin with three TMDs is depicted in this figure, following the proposal of viroporins with three TMDs. Figure adapted from [2].

Recent categorization places viroporins into two major classes based on the number of TMD that are then further classified into A or B subclasses based on their membrane topology [2]. Single TMD viroporins in subclass A have their N terminus facing the ER lumen while those in subclass B have their C-terminal tails in the ER lumen. For Class IIA and IIB viroporins, both N- and C-terminus are inside the ER lumen or the cytoplasmic matrix, respectively (Figure 1). An additional third class of viroporins may be necessary as viroporins with three-pass TMD have been proposed, such as the non-structural protein 4 (NSP4) of rotavirus [49] and 3a of severe acute respiratory syndrome SARS-CoV [31]. Due to their high structural variability under different conditions, solving the architecture of viroporins under physiological environment has been difficult. However, recent advancement in technology such as the ability to characterize protein structure at the atomic resolution using nuclear magnetic resonance (NMR) spectroscopy, has successfully resolved the structure of several viroporins [50,51,52,53]. For example, the M2 of IAV forms a tetrameric pore on the plasma membrane that adopts different conformations as it conducts proton across the membrane [54,55,56], whereas for p7 of HCV, a hexameric flower-shaped complex was revealed via single-particle electron microscopy [57,58]. p7 has also been found to exist in heptameric form using transmission electron microscopy [59] and a model of how both forms could coexist was proposed [60]. Several key residues that line the inside of the ion channel have been shown to be essential for the activation of the protein via protonation. For instance, mutating the two key histidine residues, H22 and H51 of the human respiratory syncytial virus (hRSV), SH viroporin rendered the ion channel inactive [61], which is reminiscent of the H37 residue in the M2 ion channel [62].

Viroporins have several known functions at different stages of the viral replication depending on their cellular location during the viral life cycle. While the majority of viroporins play a major role in the final viral release and budding stages, some have been proven to be essential at the early viral genome uncoating and replication steps. Table 1 gives a list of viroporins and their known roles during viral replication determined thus far. Viroporin does not form part of the viral RNA replication complex but is absolutely necessary for the pathogenesis. For instance, the absence of the Vpu viroporin in HIV-1 resulted in the retention of viral particles in the plasma membrane and thus a reduction in infectious viral particle release [63,64,65]. In addition, Vpu can also induce the degradation of CD4 at the ER to release the Env glycoprotein from the CD4/Env complex for the production of infectious particles [21,66,67,68,69]. Lastly, p7 of HCV is required for the production of infectious viral particles *in vitro* by preventing the acidification of the intracellular membrane vesicles [70] and is absolutely critical for its infection *in vivo* [71]. Due to their pivotal roles in the viral life cycle, viroporins have become the target of interest in the antiviral therapy with emphasis on HCV p7 and Vpu of HIV-1 [72,73,74,75,76]. In this review, we highlight the importance of viroporins in the viral life cycle as well as the role they play in cellular immune induction.

## 2. Viroporin and the Viral Life Cycle

### 2.1. Viral Entry and Uncoating

In order for infection to occur, viruses first have to bind to and penetrate the host plasma membrane to deliver the genetic elements into the cytoplasm for replication to take place. Enveloped and non-enveloped viruses employ different strategies to achieve the same goal (see review [77,78,79]). Upon binding of its hemagglutinin glycoprotein (HA) to the host surface sialic acid, IAV is internalized via the endosomal trafficking [80,81]. Localization of M2 at the viral membrane enables it to function as a proton channel and acidify the interior environment of the virus within the endosome [7]. Under low pH conditions, fusion of the endosomal and viral membrane leads to the release of the nucleoprotein complex from the matrix protein M1 and subsequent uncoating of the viral RNA genome in the cytoplasm [8,9,10]. The M2 homotetramer has been well characterized throughout the years [7,50,56,82,83]. The protein has been dissected into smaller sections and each has a different role in contributing to the pathogenesis of this virus [15,16,56,84]. Mutants with a defective M2 ion channel activity are still able to complete the viral life cycle in a cell culture system although they are severely compromised as compared to their wild-type counterparts. In addition, their growth was significantly suppressed when used to infect mice [85,86]. This shows that M2 is essential for the overall fitness of IAV as well as its replication efficiency in host cells. Given its role in the virulence of IAV, an inhibitor has been developed against M2 to antagonize its ion channel function. For example, the anti-influenza drug, rimantadine, is believed to bind and stabilize M2 in the closed conformation and thus renders it inactive by preventing proton conductance [50].

For the non-enveloped virus, Simian virus 40 (SV40) encodes up to three known pore forming proteins, VP2, VP3, and VP4, each of which is activated at a different stage of the life cycle with a different mechanism of action [44,46,47,48,87,88]. Upon endocytosis into host via the caveolae-1 coated vesicles [89,90], the virus is translocated to the endoplasmic reticulum (ER) where reorganization of the capsid and subsequent escape of the viral particle into the cytosol occurs. VP2 and VP3 are proposed to be responsible for the escape of the virus into the cytosol as they are able to integrate into the ER membranes [44,45,47]. Virus-like particles devoid of VP2 and VP3 failed to traverse through the ER membrane into the cytoplasmic space [45]. Consistently, mutant viruses lacking VP2 and VP3 pore-forming activities showed impaired viral infection [46]. These results suggest that VP2 and VP3 are required for the initial infection stage before the uncoating of the viral genome can take place [91].

### 2.2. Viral Replication and Assembly

Following the release of the viral genome, replication takes place in specialized membrane compartments or virus-induced inclusion bodies via reconditioning of the host intracellular machineries. IAV can perturb the trans-Golgi network (TGN) using its M2 viroporin to affect the secretory pathway in the host. In addition to its role in virus uncoating, the ion channel activity of M2 is required for the trafficking and processing of viral proteins, such as the HA glycoprotein [11] as well as M2 itself. Expression of M2 delayed its presentation at the cell surface, which could be prevented using a viroporin inhibitor, amantadine. Using its proton channel activity, M2 prevents the acidification of the trans-Golgi, resulting in delay of protein trafficking through the TGN [12]. By doing so, M2 helps to coordinate the presentation and maturation of the viral glycoprotein on the membrane with viral assembly [12,13,14].

The Vpu viroporin is only present in HIV-1 and absent from the type 2 HIV virus genome [92]. Vpu is a selective ion channel [93] that is believed to play two main functions in the viral life cycle. The cytoplasmic domain is responsible for the interaction with and degradation of CD4 while the TMD plays a role at the viral release stage, which will be discussed in the following section. During viral replication, the Env glycoprotein forms a stable complex with the cellular CD4 molecule in the ER, thus preventing the trafficking of both Env and CD4 to the cell surface [66,67,68,94,95,96]. The cytoplasmic domain of Vpu can physically bind CD4 [97,98,99] and induce its degradation [17,18,19,20]. This induction is dependent on the phosphorylation of two key residues, Ser52 and Ser56 in the cytoplasmic region of Vpu [100,101]. A key residue located in the TMD of Vpu, Trp22, though not required for the interaction between Vpu and CD4, is crucial for the targeting of CD4 to the ER-associated degradation pathway (ERAD) [102]. In the absence of Vpu, CD4 molecules are incorporated into the HIV-1 particles, causing the formation of glycoprotein gp120-CD4 complexes and reducing the level of functional Env glycoproteins on the virion surface [21].

In poliovirus, 2B and 3A viroporins can inhibit the cellular protein secretion pathway by disassembling the Golgi complex or by blocking the ER–Golgi trafficking, which results in the accumulation of membrane vesicles in the cytoplasm [33,34]. By interfering with the secretory pathway, poliovirus thwarts the host immune response against viral infection by shutting off the nascent MHC class I trafficking as well as down-regulating cytokine release [103,104]. Similarly, Coxsackievirus 2B protein can also inhibit protein trafficking through the Golgi complex by forming pores in the ER membrane, leading to efflux of Ca^2+^ ion from this compartment into the cytosol [38]. Alterations in the ion gradients are known to impact the membrane vesicle fusion and transport events [105,106,107,108]. On the other hand, release of ER calcium into the cytosol can also trigger an immune response via the activation of inflammasomes as well as autophagy, two of the mechanisms developed by the host to clear off viral infection. However, viruses have evolved to hijack these protective mechanisms to benefit their own replication. These will be discussed in the next section.

Another animal virus that manipulates the host calciomic to its own replication benefit is the diarrhea-inducing rotavirus. The hallmark of rotavirus infection is drastic changes in the calcium concentration, where ER calcium is leaked into the cytosol, activating the ER calcium sensor stromal interaction molecule 1 (STIM1), which subsequently leads to an influx of calcium through the plasma membrane and ultimately an increased in the cytoplasmic calcium level [109]. When the calcium store from ER is emptied into the cytoplasmic space, a protective calcium signaling pathway, calcium/calmodulin-dependent kinase kinase-β (CAMKK-β), is activated to initiate autophagy, a host mechanism for maintaining cellular homeostasis within the organism [110]. Rotavirus then hijacks this autophagy machinery and subverts it into a tool to transport its viral proteins to the replication site for assembly [42]. The viral protein responsible for the fluctuation of calcium store is none other than the NSP4 viroporin [49,111,112,113,114]. NSP4 is synthesized as an ER trans-membrane glycoprotein but can also exist in secreted form as enterotoxin to induce diarrhea by simultaneously activating secretion through a Ca^2+^-activated Cl^−^ channel and inhibiting absorption by the epithelial Na^+^ channel and the Na^+^/glucose co-transporter [115]. The domain responsible for the increase of cytosolic Ca^2+^ is the viroporin domain from residue 47 to 90 as mutation of this region completely abolished its effect on calcium elevation [49]. NSP4 interacts with various host factors to facilitate different stages of the viral life cycle as well as contributing to the viral pathogenesis [116,117,118,119,120]. The enterotoxin form of NSP4 binds to integrins and activates calcium mobilization [120], whereas the cytoplasmic tail of NSP4 can directly interact with the α- and β-tubulin, acting as a viral microtubule-associated protein (MAP) [117]. Since NSP4 is synthesized in the ER, the ER–Golgi intermediate compartments of the infected cells will be coated with NSP4, leading to direct attachment of these vesicular compartments to the cytoskeleton and stalls further translocation [121]. Two studies showed that silencing of NSP4 led to a more diffuse distribution of viral proteins in the cytoplasm as well as a decrease in viral yield. Furthermore, Silverstri *et al.* went on to show that NSP4 acts as the modulator of viral transcription, where suppression of NSP4 expression prevented viroplasm maturation, the site of viral genome replication and packaging, as well as excessive transcription of viral RNA during the late infection cycle [122,123]. All this evidence supports the essential role of NSP4 in the rotavirus lifecycle as a multifaceted viral protein that connects the different stages of viral replication events to ensure a productive infection.

In the family of Coronaviridae, viroporin appears to be encoded by the small envelope membrane (E) proteins [124,125,126]. Conflicting results have been obtained for the structural determination of E protein in different coronaviruses, due to their tendency to aggregate and the various conditions used to express the protein that affect the palmitoylation of the protein [127,128,129,130]. The E protein is important for the assembly and morphogenesis of the virus [29,30]. In the murine coronavirus, co-expression of the E protein and another membrane glycoprotein, M, were sufficient for the assembly and release of viral envelope particle, independent of the nucleopcapsid, an assembly process that is unlike many other enveloped viruses [28]. When mutations were made in the E gene, the overall fitness of the mutant viruses were significantly reduced as compared to the wild type [131]. Future studies are needed in order to fully decipher the mechanism of viroporin E in the assembly and egress of the virus.

Besides acting as an ion channel, viroporin can have important roles that are independent of its pore-forming and ion-conducting abilities. Recent data have provided strong evidence for defining the role of p7 in the viral life cycle of HCV. The assembly of HCV viral particles commences in the cellular organelle termed the lipid droplets (LD) [132] (see recent review [133] for a detailed overview of the HCV life cycle). During the viral assembly process, LD is proposed to serve as a platform for viral assembly by concentrating the viral capsid core protein in close proximity to the replication complex located in the ER membranes [134,135]. p7 has been shown to interact with three other HCV structural proteins, including the core protein and two other glycoproteins E1 and E2 [136]. A study by Gentzsch *et al.* showed that mutations in the p7 cytoplasmic loop led to retention of core in the LD and subsequent defects in virus production [26]. Besides interaction with the structural proteins, p7 can also affect the processing of the viral polyprotein between E2/p7 and p7/NS2; without it there is defective virus production [27,137]. Another important role that p7 has in the HCV life cycle is manipulating the distribution of the non-structural protein NS2 and influencing the interactions between NS2 and other viral proteins to coordinate the assembly process, independent of its ion channel activity [24,25,137]. All these imply that in addition to its calcium channel activity [138], p7 plays an important part in the morphogenesis of the HCV virion. Thus it is of no surprise that p7 has emerged as the target of antiviral therapy for HCV. Several p7 inhibitors have been found, such as the M2 inhibitor amantadine, and iminosugars, and the hunt for more inhibitors is still actively pursued [74,76,139,140].

### 2.3. Viral Release

The majority of the viroporins are important in the final stage of the viral life cycle because they facilitate the release of fully assembled virus particles from the host. In the non-enveloped virus, lysis of the host cells is commonly used to release infectious virions devoid of host membrane. In enteroviruses, the non-structural protein 2B forms pores in ER membrane, leading to leakage of calcium into the cytoplasm. Disruption of calcium homeostasis activates several host immune responses such as autophagy, which ultimately leads to cellular apoptosis (see review [41]). For example, in Coxsackievirus, 2B has been found to localize to the ER and plasma membrane at different stages of the infection. A model has been proposed whereby 2B alters the permeability of the ER and plasma membrane to facilitate viral release by affecting the calcium content [39]. Subsequent viral release is most likely achieved through apoptosis induction via the calcium dependent mitochondrial pathway [40,41]. In Poliomaviruses such as SV40, proliferation occurs in the nuclei and release of infectious particles occurs following cells’ rupture [88]. VP4 of SV40 induces membrane perforation by forming toroidal pores, fusing the outer and inner leaflets of the lipid bilayers to initiate viral release. Mutational analysis indicated that Pro70 located within the hydrophobic region is essential for the membrane disruption ability of VP4 [47].

For enveloped viruses, budding and scission are commonly used to release the virus from infected cells. The 6K viroporin of the alphavirus [141] appears to assist in viral release via direct insertion into the plasma membrane. Through insertion into the lipid bilayer, it is proposed that the interfacial domain of 6K can then disrupt the cohesion of the outer membrane phospholipid to prompt viral release [35,37]. Deletion of the 6K gene, as well as mutational analysis of the conserved amino terminal region of 6K essential for its partitioning into the membrane, led to a defect in the budding of infectious particles [36,37], further supporting the role of viroporin in the release of alphavirus. In IAV, M2 can induce membrane fission using a conserved amphipathic domain within M2, residues 47–61, which bind cholesterol and induce membrane curvation, leading to invagination of the liposome membrane and subsequent pinching off of the membrane vesicles [15,16]. Deletion of this region resulted in a defect in viral release where a phenotypic beads-on-a-string budding pattern is seen due to incomplete scission, further substantiating the direct role of M2 in virus budding [16,142,143]. In SARS-CoV, in addition to the E protein, a second viroporin, 3a, was identified [31]. Depletion of 3a using siRNA significantly suppresses the release of viral particles into the culture media [32]. However, the underlying molecular mechanism involved is yet to be determined.

In addition to targeting CD4 for degradation, the ion channel Vpu is also required for efficient release of HIV-1 virions from infected cells, where it assists in depolarizing the host membrane and thus disrupting the electrical barrier at the membrane to enhance the release of HIV-1 particles [23]. Similar to M2, different regions of Vpu can interact with different host factors and contribute to the viral pathogenesis [22,144,145]. For instance, the second alpha-helix within Vpu can interact with tetherin, targeting it for endosomal degradation to counteract its inhibitory effect on viral release [145,146,147,148,149] and at the same time suppressing the innate immune response to ensure a successful infection [150,151]. A recent study also found that the binding of Vpu H2 α-helix within the cytoplasmic tail to tetherin is able to displace the latter from the viral assembly site at the cell surface in order to produce virions devoid of tetherin on the surface [152]. In the absence of a functional Vpu, mature virions accumulate in the endosomal compartments or remain attached to the cell surface by tetherin [146,150,153]. A crystal structure of a protein complex containing both Vpu-tetherin as well as the core of the clathrin adaptor protein complex 1 (AP1) was obtained recently and this opens up the possibility that Vpu may be able to modulate the fate of other host proteins by hijacking the clathrin-dependent trafficking pathways [154].

## 3. Viroporin-Induced Host Response

During an active infection, a virus hijacks the host molecular machinery and turns it into a virus factory. When the ion channel activity of viroporin is activated during viral infection, perturbation of the host membrane permeability occurs, leading to disruption of the cellular ion homeostasis and subsequent cytophatic events. Many viroporins are expressed as ER proteins and remained localized to the ER during virus replication, while several have been shown to be partially localized to the mitochondria [155,156,157]. The ER serves as the organelle that stores a major portion of the cell’s calcium ion where a >1000-fold gradient is maintained across the ER membrane and the cytoplasm [40,158]. Expression of viral ion channels at the ER membrane causes leakage of Ca^2+^ from the ER storage into the cytoplasm (see review [40]). Fluctuation of the cytosolic Ca^2+^ concentration can inhibit protein trafficking through TGN and inhibit antiviral response, as mentioned in the previous section. It can also trigger several defensive signaling pathways, including apoptosis, autophagy, and inflammasome formation, in an attempt to contain and eliminate the invader. However, viruses have evolved to adapt and subvert these defense mechanisms for their own growth benefits. The strategy involved and how these antiviral pathways become pro-viral growth are only beginning to be explored.

### 3.1. Apoptosis/Autophagy

Apoptosis is a genetically programmed mechanism used by the host to eliminate damaged or unwanted cells through the activation of caspase cascade. There are two main signaling pathways that can activate programmed cell death, the extrinsic or receptor-mediated pathway, and the intrinsic or mitochondrial pathway [159,160]. Perturbation of the ER Ca^2+^ storage can also activate an ER-specific apoptotic pathway that ultimately leads to the activation of the common apoptosis effector caspase, caspase-3 [161]. Viroporins from several viruses have been shown to trigger apoptosis in a caspase-dependent manner but the mechanism involved differs between each virus. HCV p7 has been shown to induce apoptosis via both the intrinsic and extrinsic pathways. In addition, mutational analysis indicated that such ability is independent of its ion channel activity [162]. The HIV-1 Vpu protein also induces caspase-dependent apoptosis but it does so by preventing the activation of NF-κB and thereby suppressing the expression of several NF-κB-dependent anti-apoptotic genes, such as Bcl-xL, a member of the Bcl-2 family [163,164]. Recent work by Madan *et al.* showed that expression of several viroporins from RNA viruses including 6K of Sindbis virus, M2 of IAV, 2B and 3A of poliovirus, p7 of HCV, and E protein of mouse hepatitis virus A59, are able to activate caspase-3 and lead to the release of cytochrome c from the mitochondria [156], proving the ability of these viroporins to activate the intrinsic programmed cell death pathway. The secreted form of NSP4 can also activate the intrinsic pathway by translocating to the mitochondria and interacting with the mitochondrial integral proteins, the outer mitochondrial membrane pore (VDAC), and the adenine nucleotide translocase (ANT), leading to the release of cytochrome c and depolarization of mitochondria [157].

Another cell death pathway that is often activated during an insult is autophagy. Autophagy occurs through the intracellular membrane trafficking system by delivering damaged or unwanted cellular material from the cytosol to the lysosome for degradation, and thus can be referred to as “self-eating” [165]. During autophagy, cellular components are sequestered into a double-membrane vesicle termed an autophagosome, which then fuses with a lysosome where degradation can occur [166,167]. Viruses have evolved to manipulate and interfere with different stages of this destructive process to their benefit. For instance, M2 is able to inhibit the fusion between autophagosome and lysosome independent of its proton channel activity by interacting with the autophagy-related protein, Atg6/Beclin-1 [162,168,169]. By doing so, degradation of the autophagosomes is inhibited, leading to accumulation of these vesicles in the infected cells. Some viral infections can induce autophagy-related vesicles’ formation so they can be subverted to the viruses’ advantage. During poliovirus infection, formation of autophagy-like double-membrane vesicles is induced by 2BC and 3A viral proteins [170,171,172]. The 3A viroporin then further inhibits the movement of these vesicles along the microtubules and blocks the maturation and degradation of these vesicles to benefit the viral growth and release [173,174]. For rotavirus infection, autophagy is induced when NSP4 permeabilizes the ER membrane and releases the calcium storage into the cytoplasm. A sudden surge in cytoplasmic calcium concentration activates the calcium-dependent autophagy pathway, which is then tricked into transporting the viral proteins from the ER to the viroplasm for replication and assembly [42,43,114].

### 3.2. Inflammasome Activation

Another antiviral innate immune response that is activated during ion flux is the complex termed the “inflammasome.” An inflammasome is a caspase-activating complex that upon induction results in caspase-1 activation and pro-inflammatory cytokines secretion [175]. There are several distinct inflammasomes that can be activated via different viral factors and pattern recognition receptors (PRR) [176]. One of the inflammasome complexes that can be activated via disturbances in intracellular ionic concentration in addition to the pathogen-associated molecular patterns (PAMPs) is the Nod-like receptor family, pyrin domain-containing 3 (NLRP3) inflammasome [177,178]. Several respiratory viruses are known to activate NLRP3 inflammasome in the lung during infection by causing disturbances to the cellular potassium and calcium ion homeostasis via viroporins [179]. IAV is the most common activator of the NLRP3 inflammasome, in which it induces the maturation of caspase-1 via dsRNA [180] and subsequent secretion of pro-inflammatory cytokine, such as IL-1β [181,182]. Activation of NLRP3 inflammasome requires two signals [183]. In addition to the dsRNA, M2 provides the second signal by altering the ionic concentration within the TGN via its proton channel activity [184]. The hRSV SH viroporin also induces NLRP3 inflammasome activation in a similar manner. Wild-type hRSV induces caspase-1 maturation and IL-1β secretion, which can be prevented using a SH-defective mutant or by treating the cells with ion channel inhibitors, emphasizing the importance of SH ion channel activity in inflammasome activation [185]. The Encephalomyocarditis virus (EMCV), a member of the *Picornaviridae*, encodes a viroporin, 2B, similar to the poliovirus 2B viroporin [186]. Expression of 2B alone is sufficient to induce the efflux of calcium from the ER into the cytoplasm, which is a prerequisite for the activation of inflammasome and subsequent IL-1β secretion. Treatment with the calcium ion chelator BAPTA-AM completely inhibited the release of IL-1β, suggesting that 2B is solely responsible for EMCV-induced inflammasome activation [187].

## 4. Conclusions

Given that viroporins have important roles in various stages of the viral life cycle, as well as their structural differences from the human ion channels, they have become the target of interest for inhibitory drug development and antiviral therapy. The first inhibitory drug developed against M2 proton channel activity, adamantane compounds (amantadine or rimantadine), was used to treat influenza infections [188,189,190]. Since then, additional viroporin inhibitors have been discovered against other viruses, such as the latest compound, BIT225, targeting the Vpu of HIV-1 [75,191], which was initially identified from a screen for HCV p7 inhibitor [192]; the long-alkyl-chain iminosugar (*N*N-DNJ) [72,74,139] and hexamethylene amiloride (HMA) [73,139] against HCV p7; and pyronin B against hRSV SH protein [193]. In addition, different modes of inhibition against viroporins of other viruses have been identified from a single drug. For instance, amantadine (and its derivatives) has been shown to have an inhibitory effect not only on its intended target, M2 [189,190], but also on the channel conductance of HCV p7 [70,74,138,155,194], using a different mechanism of inhibition [58] from M2 [195]. Since viroporin is an important virulence factor of the virus, viroporin drug-resistant mutants have emerged after amantadine was used as a treatment for IAV infection [189,196,197,198], further emphasizing the need to synthesize a better viroporin inhibitor. As the structure of the viroporin determines how a drug can bind and exert its inhibition, resolving the unique structure of each ion channel is of the essence in developing novel antiviral drugs. By studying the structures of the drug-resistant M2 mutants, Pielak and colleagues were able to determine the mechanisms of inhibition of the M2 inhibitors as well as the mechanism of resistance conferred by the mutations [7,50,51]. This information would be useful for the future development of viroporin inhibitors with improved binding and mechanism of inhibition. A recent paper by Foster *et al.* discussed the feasibility of using structure-guided design in search of new compounds with high binding affinity against HCV p7 [76]. Thus, solving the architecture of viroporins together with better characterization of their functional aspects will certainly aid in understanding the mechanism of action of each unique viroporin and help in developing therapeutics against these viral membrane channels. In addition to its role as an ion channel, viroporin often interacts with numerous cellular factors or signaling pathways for viral morphogenesis and assembly. Synthesizing specific inhibitors or mimetic peptides that can block such interaction will certainly be useful for fighting and developing a cure for viral infection.
